# Approval processes in evidence-based clinical practice guidelines sponsored by medical specialty societies

**DOI:** 10.1371/journal.pone.0229004

**Published:** 2020-02-12

**Authors:** Jeffrey Sonis, Olivia M. Chen

**Affiliations:** 1 Department of Social Medicine, University of North Carolina at Chapel Hill, Chapel Hill, North Carolina, United States of America; 2 Department of Family Medicine, University of North Carolina at Chapel Hill, Chapel Hill, North Carolina, United States of America; 3 Department of Dermatology, University of Michigan, Ann Arbor, Michigan, United States of America; Universitat Witten/Herdecke, GERMANY

## Abstract

**Objective:**

To determine the approval processes for evidence-based Clinical Practice Guidelines sponsored by medical specialty societies in the United States.

**Study design and setting:**

Cross-sectional analysis of published Clinical Practice Guidelines and Guideline procedure manuals, sponsored by the 43 members of the Council of Medical Specialty Societies in the United States. Approval processes were measured by written evidence in the specialty society’s guideline procedure manual or published guidelines, through May 2017.

**Results:**

Among the 36 (of 43) specialty societies that published evidence-based Clinical Practice Guidelines, 27 (75%) required approval by a committee representing the society as a whole. None specified the criteria used for approval decisions. Six specialty societies (17%) required approval but included procedures to maintain some editorial independence for the guideline development group, such as approval by a guideline committee not an executive committee or approval dependent on fidelity to established guideline methodology, not content. One society required Board review, but not approval. The approval process was not reported by 2 (6%) of the specialty societies.

**Conclusions:**

Most medical specialty societies in the U.S. require approval of guidelines by a board that represents the society as whole. Since medical specialty societies have loyalties to the patients they serve and to their physician members, and because the interests of those two groups may differ, such an approval process introduces a potential conflict of interest into the guideline development process.

## Introduction

There is widespread agreement that conflict of interest has the potential to impair the ability of guideline development panels to draw valid conclusions and make sound recommendations that are worthy of patient, physician and policy-maker trust [1,2]. Most of the research and commentary on conflict of interest in the guideline development process has focused on panel member financial and intellectual conflicts of interest and on conflicts related to guideline funding sources that have a financial stake in tests or treatments that may be recommended [3,4].

However, there has been little attention directed to the potential for the policies and procedures of a guideline development panel to introduce conflict of interest. This issue is particularly salient in practice guidelines that are sponsored by medical specialty societies.

Medical specialty societies produce most clinical practice guidelines (CPGs) used in the United States [5]. Those societies possess advantages for the creation of CPGs, including access to clinicians with relevant expertise, members likely to be supportive of guideline development, and funding to support guideline development [5,6]. However, specialty societies also have the dual obligations of advocacy for the patients served by that specialty as well as the professional interests of their members [7,8]. Since the financial and professional interests of clinicians may conflict with the health needs of patients in certain situations, this dual mission raises the concern for conflict of interest when specialty societies create evidence-based CPGs [5].

The potential conflict of interest due to dual missions by medical specialty societies might be manifest through selection of guideline panel members whose published work or public actions suggest a particular position favorable to the professional interests of the specialty society. However, the National Academy of Medicine (previously known as the Institute of Medicine, IOM) proposals for minimizing individual-level conflict of interests should, at least in theory, reduce the probability of that type of conflict of interest [1].

The potential for conflict of interest due to dual loyalties by specialty societies could also be introduced if the editorial independence of the guideline development panel were diminished or compromised in any way. Editorial independence is an important component in the production of high-quality guidelines because it guarantees that the views of the guideline panel, which are intended to reflect only evidence and patient preference, are not influenced by others with potentially competing interests. The AGREE II instrument for assessing guideline methodological quality includes the following item in its “editorial independence” domain: “the views of the funding body have not influenced the content of the guideline” [9].

In this paper, we focus on one key element in the guideline development process that may foster or inhibit editorial independence of guideline panels sponsored by medical specialty societies: final approval. Specialty societies certainly have legitimate reasons to make sure that a guideline accurately reflects the evidence and offers appropriate recommendations before it is published with the specialty society’s imprimatur. However, if a specialty society introduces an approval process that requires a guideline to be approved by a committee or board that represents the society as a whole (e.g., the Executive Committee or Board of Directors), editorial independence may be compromised.

The purpose of this project was to determine the characteristics of the approval process for evidence-based CPGs created by the 43 medical specialty societies that are members of the Council of Medical Specialty Societies (CMSS), a group representing 790,000 U.S. physicians [10]. Specifically, we assessed whether final approval of CPGs produced by those specialty societies was required and, if so, whether that approval was granted by a committee representing the society as a whole. We also assessed the criteria for approval.

## Materials and methods

Between October 2016 and March 2017, two reviewers (J.S., O.C.) independently assessed whether each of the 43 medical specialty societies in the CMSS [10] produced evidence-based CPGs by reviewing CPGs identified through a search of the specialty society web site. Because each specialty society organized its web site differently, we searched all sections (and all subsections, if necessary) of each specialty society web site to find clinical practice guidelines and guideline development manuals, if available. S1 Table in the Supporting Information shows the URL for each medical specialty society and the section of each society’s web site that included clinical practice guidelines and a guideline development manual, if it existed. If guidelines were not found after searching all sections of a medical specialty society web site, we searched the web site, using the search tool on that web site using the following terms: clinical practice guideline, practice guideline, guideline, practice parameters, practice bulletins. We also reviewed guideline synopses available for each medical specialty society, if available, on the National Guideline Clearinghouse (NGC) website [11]. (The web site for the NGC is no longer online; its funding from the Agency for Health Care Research and Quality ended on July 16, 2018 [12]. The guideline synopses that were on their web site are no longer publicly available.) We did not search Google or other general search engines for specialty society guidelines or guideline development manuals.

A CPG was considered evidence-based if the evidence used by the guideline panel was based on a systematic review and there were explicit ratings of the quality of the evidence [1]. A guideline that reported recommendations without specifying that a systematic review served as the evidence base or without explicit ratings of the quality of the evidence was considered to not be evidence-based for the analyses in this study. A medical specialty society was considered as a producer of evidence-based CPGs if at least one of its guidelines met those criteria. If it was unclear, based on the criteria described, whether the available guidelines produced by a specialty society were evidence-based, we categorized the society’s guidelines as uncertain. Societies that produced joint evidence-based guidelines with other specialty societies, but did not produce any guidelines on their own, were considered as producers of evidence-based guidelines. Societies that endorsed or affirmed evidence-based guidelines produced by other societies but were not involved in the creation of those guidelines were considered to not be producers of evidence-based guidelines.

To determine the process of approval for evidence-based CPGs produced by each specialty society, each reviewer first searched the specialty society’s website for CPG development procedure manuals. If procedure manuals were identified, the structure of the final approval process was evaluated using that procedure manual. If procedure manuals were not identified or if the procedure manual did not specify the approval process, each reviewer searched for descriptions of the guideline approval process in individual guidelines and in their structured synopses, available on the National Guideline Clearinghouse website. We searched the most recent guidelines first and continued the search backward in time until we identified a guideline that included information about the approval process, if any. S2 Table in the Supplementary Information shows the specific guideline (or guideline development manual) that we used as the basis for the decision about the approval process.

For those specialty societies that produced evidence-based guidelines, we evaluated the following components of the approval process:

1. Was there a required process of CPG approval?
2. If approval was required:
  - Which committee granted approval?
    1. Governing body representing the society as a whole (e.g., Board of Directors, Executive Committee, etc.)
    2. Other committee, such as a Guidelines Committee
  - What criteria were used for approval decisions?

We also assessed whether, among those specialty societies that required guideline approval, there was an opportunity for review of the guideline by individuals and/or groups other than those that produced the guideline, prior to the approval process. Differences between the two reviewers were resolved by consensus, after reviewing the relevant web sites and documents together.

Descriptive statistics are reported. Confidence intervals are not reported because we included the universe of medical specialty societies in the Council of Medical Specialty Societies in the United States rather than a sample of those societies. Inter-rater reliability was assessed with the unweighted kappa statistic [13].

This research was exempt from Institutional Review Board review because only publicly available information was collected.

## Results

All 43 members of the CMSS produced clinical recommendations but only 36 of them produced at least one evidence-based CPG, as of March 1, 2017. There was 93% agreement between the two raters in the assessment of whether a specialty society produced evidence-based guidelines, kappa 0.77. Of the 36 specialty societies that produced evidence-based CPGs, 27 (75%) required approval by a governing body that represented the society as a whole without specification of the criteria used for approval decisions, 7 (19%) implemented procedures to maintain some editorial independence for the guideline panel and 2 (6%) did not provide sufficient evidence to determine whether their CPGs required approval (Table 1). S2 Table in the Supporting Information shows characteristics of the approval process, if any, for each medical specialty society in the CMSS. There was 95% rater agreement in the assessment of which committee, if any, approved a guideline, kappa 0.81.

**Table 1:** Approval requirements for evidence-based guidelines created by medical specialty societies.

	No. (%) (n = 36)
Required	
Content approved by governing committee (e.g., Board of Directors, Executive Committee)	27 (75%)
Content approved by other committee (e.g., guideline committee)	3 (8%)
Content approved by Board with stipulation*	1 (3%)
Fidelity to guideline process, but not content, approved by Board of Directors	2 (6%)
Not required	
Reviewed but not approved by Board	1 (3%)
Approval not specified	2 (6%)

^*^Edits included only when “substantiated by a preponderance of appropriate level evidence”^17^

Nineteen (70%) of the 27 specialty societies that required governing body approval without specifying the criteria for approval used a review process that incorporated opportunities for evaluation of the guideline by persons and groups other than those that produced the guideline, prior to approval. These opportunities included feedback from specialty society members, feedback from external peer reviewers and evaluation by special review committees within the society.

Of the seven specialty societies that included some editorial independence for guideline panels, two (American Society of Colon and Rectal Surgeons, Society of Gynecologic Oncology) required approval by a committee that oversaw guideline development [14,15] and one (American Society for Clinical Pathology) required approval by a “Special Review Panel” [16]; none of those three committees represented their entire society. One specialty society (North American Spine Society) required Board approval, but noted that edits were considered “only when substantiated by a preponderance of appropriate level evidence” [17]. Two (American Academy of Otolaryngology-Head and Neck Surgeons, American Gastroenterological Association) required Board approval, yet specified that approval should be based on fidelity to approved guideline development methodology not content [18,19]. One (American College of Occupational and Environmental Medicine) required that guidelines be reviewed, but not approved, by the Board [20]. Details on those approval processes are shown in S2 Table in the Supporting Information.

## Discussion

Of the 36 medical specialty societies that produced evidence-based CPGs, 27 (75%) required approval by a group that represented the society as a whole, without specifying the approval criteria. Because medical specialty societies represent the interests of their members in addition to patient interests, this approval procedure introduces the potential for conflict of interest into a process designed to promote recommendations based solely on evidence and patient preferences. This approval procedure also appears to raise an opportunity for a conflict with a key provision regarding editorial independence in the AGREE II checklist (designed to assess the methodological quality of CPGs): “the views of the funding body have not influenced the content of the guideline” [9]. The structure and criteria for final approval of CPGs produced by medical specialty societies is important because such guidelines may recommend specific tests or treatments that have both financial and health implications.

To be clear, we have no specific evidence that conflict of interest has affected any evidence-based CPG produced by any specialty society. However, it is not difficult to envision realistic situations in which the requirement for approval of an evidence-based guideline by a committee representing the medical specialty society as a whole could influence the content of a guideline, particularly if no criteria for approval decisions had been codified. The conflict of interest could even arise prior to the approval process itself if the guideline development panel knew that Board approval would be required for guideline publication in the medical society’s official journal. Such knowledge could influence, even unwittingly, guideline content. Further, a board could require that a guideline be revised if the recommendations conflicted with the economic interests of its members. Empirical data on the impact of these types of potential conflicts of interest on actual guidelines are needed, but will be difficult to obtain.

Some medical societies vest approval with committees that do not represent that society as a whole. Two medical specialty societies (American Society of Colon and Rectal Surgeons, Society of Gynecologic Oncology) required approval by a committee that was overseeing guideline development and one (American Society for Clinical Pathology) required approval by a “Special Review Panel”. We believe that separating approval from a committee that represents the society as a whole introduces a degree of editorial independence that may reduce the potential for conflict of interest.

Similarly, having explicit criteria based on adherence to pre-approved methodological standards, as done by the American Academy of Otolaryngology-Head and Neck Surgeons and the American Gastroenterological Association may reduce the potential for conflict of interest even though the power of approval for those societies still rests in the hands of their respective Boards.

The fact that most medical specialty societies require Board approval does not mean that those societies intend to introduce conflict of interest. Indeed, it is reasonable for a specialty society to want to have some sort of safeguard to prevent a panel from putting out a guideline in the name of the society with recommendations that are inconsistent with evidence, impractical or incapable of being implemented in practice. It is not inconceivable that a guideline development panel could run off the rails and it is not just reasonable but important for a medical specialty society to prevent such guidelines produced in their name from being published.

The focus on the approval process in this project is not meant to imply that the guidelines that are developed by specialty societies are approved by the governing body as written without opportunity for peer review or revision. As noted above, 70% (i.e., 19) of the 27 societies that required governing body approval included opportunities for feedback and revision prior to approval. Moreover, it is possible that the other 30% (i.e., 8) specialty societies, of the 27 that required governing body approval, included review processes but did not describe those processes in either a guideline procedure manual or in published guidelines. Opportunities for peer review and revision might reduce the potential for conflict of interest, particularly if the peer review is conducted by reviewers who are not members of the specialty society that created a guideline, but they do not eliminate that possibility. Indeed, peer reviewers who are members of a specialty society that produced a guideline could be influenced by the required governing body approval in the same way that members of a guideline development panel could be influenced. Finally, unless rules for the peer review process are specified a priori, changes recommended by a peer review group that are designed to maintain fidelity to evidence or approved methodology could be ignored by the group vested with power of approval.

So how can medical specialty societies that produce evidence-based guidelines prevent poor guidelines from being put out in their name without introducing conflict of interest by requiring Board approval?

One solution would be to have government agencies instead of medical specialty societies produce CPGs. By taking medical specialty societies out of the guideline equation, the potential for financial conflict of interest by the society itself would be eliminated (though conflict of interest by individuals would be unchanged). However, government sponsorship of guidelines also has the potential to introduce conflict of interest when scientific evidence conflicts with governmental political or ideological goals [21]. Government-sponsored guidelines should not be considered immune from potential conflict of interest.

Another solution would be for specialty societies to adopt approval processes similar to those used by the seven specialty societies that maintain some degree of editorial independence of the guideline development panel. Options include:1) having the approval process run by a committee separate from the governing body of the specialty society; 2) requiring that edits recommended by a review committee be based on evidence; 3) basing approval on fidelity to standard guideline methodology and process but not content.

To obtain complete editorial independence, the review and approval processes would need to be conducted by an external committee, composed of patients and methodological and content experts who are not members of the specialty society, based on explicit and standard criteria, focusing on the degree to which the guideline development process followed methodological standards, such as those proposed in the IOM Report, Guidelines We Can Trust [1]. It may be difficult, though, to identify content experts who are not members of the specialty society. In addition, specialty societies are unlikely to be willing to relinquish the power of approval to an external group.

The guideline approval process used by the World Health Organization (WHO) may be a useful model for maximizing editorial independence while recognizing that specialty societies may be unlikely to cede approval to a completely external committee. In the WHO process, final approval is determined by the Guidelines Review Committee, which is composed of internal WHO members and external methodological and content experts. Approval is based on adherence to pre-approved methodological processes. This model combines the concept of vesting a committee separate from the Board with the power to approve a guideline and the concept of basing approval on fidelity to pre-approved methodological criteria. It also adds the element of inclusion of members external to the organization on the committee that approves the guideline.

This study has several limitations. First, because we did not review every guideline produced by each society, it is possible that we failed to capture differences in guideline approval procedures for different guidelines produced by the same society. However, we did not identify a single instance of difference in approval processes across many different guidelines produced by the same societies that we did review.

A second limitation is that it is possible that one or more of the seven specialty societies that we classified as not producing evidence-based guidelines were misclassified, due to underreporting of the elements that we required to classify a guideline as evidence-based. We believe that is unlikely, though, because specialty societies that had used an evidence-based process rather than one that was only based on consensus would have been likely to report that, given the greater cachet attached to guidelines that are evidence-based.

Third, it is possible that some medical societies have incorporated procedures into the guideline development process designed to maintain editorial independence but did not report them in either a guideline manual or in published guidelines. We chose to measure guideline approval processes solely through publicly available sources on medical specialty society web sites because we believed that those societies would be unlikely to release internal documents. The disadvantage of this measurement approach, though, is that our estimate of the percentage of specialty societies that approved guidelines without specifying the criteria for approval may be an overestimate.

Fourth, we reviewed specialty society guidelines and approval manuals completed through May 2017. If specialty societies have changed their guideline approval processes in the interim, our findings may not be applicable to current approval processes.

In summary, we found that most medical specialty societies included guideline approval processes that had the potential to compromise editorial independence, thus introducing the potential for conflict of interest into a process that is designed to produce recommendations based exclusively on evidence and patient preferences. Seven specialty societies included approval processes that maintained at least some editorial independence and those processes, or ones like the WHO’s, may be useful models for all medical specialty societies that produce evidence-based guidelines.

## Supporting information

S1 Table Web sites of medical specialty societies in the council of medical specialty societies searched for clinical practice guidelines and guideline development procedure manuals. (DOCX)

S2 Table Approval processes for evidence-based clinical practice guidelines by medical specialty societies in the council of medical specialty societies. (DOCX)
