# Supplementary material for: Approval processes in evidence-based clinical practice guidelines sponsored by medical specialty societies
Source: PLoS One. 2020 Feb 12;15(2):e0229004. doi: 10.1371/journal.pone.0229004 (PMC7015697; doi:10.1371/journal.pone.0229004)
Supplement: S1 Table — (DOCX) [file pone.0229004.s001.docx]

**S1 Table. Web Sites of Medical Specialty Societies in the Council of Medical Specialty Societies Searched for Clinical Practice Guidelines and Guideline Development Procedure Manuals**

| **Medical Specialty Society** | **Uniform Resource Locator (Web Address)** | **Section of Web Site for Clinical Practice Guidelines^a^** |
| --- | --- | --- |
| American Academy of Allergy, Asthma & Immunology | https://www.aaaai.org/ | Practice Resources > Statements and Practice Parameters > Practice Parameters and Other Guidelines |
| American Academy of Dermatology | https://www.aad.org/ | Member > Clinical Guidelines |
| American Academy of Family Physicians | https://www.aafp.org/home.html | Patient Care > Clinical Practice Guidelines |
| American Academy of Hospice and Palliative Medicine | http://aahpm.org/ | No Clinical Practice Guidelines created by the American Academy of Hospice and Palliative Medicine identified in the following sections: Membership, Education & Practice, Advocacy, Career Development, About, Store. |
| American Academy of Neurology | https://www.aan.com | Policy & Guidelines > Guidelines |
| American Academy of Ophthalmology | https://www.aao.org/ | Clinical Education > Guidelines > Browse All Guidelines |
| American Academy of Otolaryngology—Head and Neck Surgery | https://www.entnet.org/ | Guidelines |
| American Academy of Pediatrics | https://www.aap.org/en-us/Pages/Default.aspx | Quality Improvement > Clinical Practice Guideline Manual |
| American Academy of Physical Medicine and Rehabilitation | https://www.aapmr.org/ | Quality & Practice > Evidence-based Medicine > Clinical Practice Guidelines^b^ |
| American Association of Clinical Endocrinologists | https://www.aace.com/ | Disease State Resources > (Separate sections for Adrenal, Bone and Parathyroid, Diabetes, Lipids and CV Health, Nutrition and Obesity, Pituitary and Neuroendocrine, Reproductive and Gonad, Thyroid. Clinical Practice Guidelines shown in each separate disease state section. |
| American College of Cardiology | https://www.acc.org | Guidelines |
| American College of Emergency Physicians | https://www.acep.org/ | Practice > Patient Care > Clinical Policies |
| American College of Medical Genetics | https://www.acmg.net/ | Practice Resources > Practice Guidelines^c^ |
| American College of Obstetricians and Gynecologists | https://www.acog.org/ | Clinical Guidance & Publications > Practice Bulletins |
| American College of Occupational and Environmental Medicine | https://acoem.org/ | Practice Guidelines: Defining Excellence |

**S1 Table. Web Sites of Medical Specialty Societies in the Council of Medical Specialty Societies Searched for Clinical Practice Guidelines and Guideline Development Procedure Manuals (cont.)**

| **Medical Specialty Society** | **Uniform Resource Locator (Web Address)** | **Section of Web Site for Clinical Practice Guidelines^a^** |
| --- | --- | --- |
| American College of Physicians | https://www.acponline.org/ | Clinical Information > Clinical Guidelines & Recommendations |
| American College of Preventive Medicine | https://www.acpm.org/ | Education & Events > Education > Practice Guidelines^c^ |
| American College of Radiology | https://www.acr.org/ | Clinical Resources > Practice Parameters |
| American College of Rheumatology | https://www.rheumatology.org/ | Practice & Quality > Clinical Support > Clinical Practice Guidelines |
| American College of Surgeons | https://www.facs.org/ | Clinical Practice Guidelines identified in multiple different sections of the website, identifiable by searching “Clinical Practice Guideline” and “Practice Guideline” in the search box on the web site. |
| American Epilepsy Society | https://www.aesnet.org/ | Clinical Resources > Guidelines |
| American Gastroenterological Association | https://www.gastro.org/ | Guidelines |
| American Geriatrics Society | https://www.americangeriatrics.org/ | Publication & Tools > AGS Guidelines, Recommendations, & Position Statements |
| American Medical Informatics Association | https://www.amia.org/ | No Clinical Practice Guidelines created by the American Medical Informatics Society were identified in the following sections: About AMIA, Membership, News and Publication, Programs, Education, Meetings & Events, Public Policy, Career Center |
| American Psychiatric Association | https://www.psychiatry.org/ | Practice > Practice Guidelines |
| American Society of Anesthesiologists | https://www.asahq.org/ | Standards & Guidelines > Practice Guidelines |
| American Society of Clinical Oncology | https://www.asco.org/ | Research & Guidelines > Guidelines, Tools & Resources > Guideline Methodology > ASCO Guidelines Methodology Manual |
| American Society for Clinical Pathology | https://www.ascp.org/content | Get Involved > Make Your Voice Heard > Guidelines and Resources > Clinical Practice Guidelines and Resources > Evidence-based Guidelines Developed |
| American Society of Colon and Rectal Surgeons | https://www.fascrs.org/ | Physicians > Clinical Practice Guidelines |
| American Society of Hematology | https://www.hematology.org/ | Education > For Physicians > ASH Clinical Practice Guidelines |

**S1 Table. Web Sites of Medical Specialty Societies in the Council of Medical Specialty Societies Searched for Clinical Practice Guidelines and Guideline Development Procedure Manuals (cont.)**

| **Medical Specialty Society** | **Uniform Resource Locator (Web Address)** | **Section of Web Site for Clinical Practice Guidelines^a^** |
| --- | --- | --- |
| American Society of Nephrology | https://www.asn-online.org/ | No Clinical Practice Guidelines created by the American Society of Nephrology were identified in the following sections: Education, Training, Pubs, Advocacy, Grants, Members, More. |
| American Society of Plastic Surgeons | https://www.plasticsurgery.org/ | Quality Resources > Evidence-Based Clinical Practice Guidelines |
| American Society for Radiation Oncology | https://www.astro.org/ | Patient Care and Research > Guidelines and Consensus Documents |
| American Society for Reproductive Medicine | https://www.asrm.org/ | For Health Professionals > News & Publications > Practice Committee Documents |
| American Urological Association | https://www.auanet.org/ | Education > Guidelines and Policies > Clinical Guidelines |
| Congress of Neurological Surgeons | https://www.cns.org/Default.aspx | Guidelines |
| North American Spine Society | https://www.spine.org/ | Research & Clinical Care > Quality Improvement > Clinical Guidelines |
| Society of Critical Care Medicine | https://www.sccm.org/Home | Research/Quality > Guidelines |
| Society of Gynecologic Oncology | https://www.sgo.org/ | Clinical Practice > Guidelines |
| Society of Hospital Medicine | https://www.hospitalmedicine.org/ | No Clinical Practice Guidelines created by the Society of Hospital Medicine were identified in the following sections: Membership, Events, Professional Development, Clinical Topics, Practice Management, Policy and Advocacy, About SHM. |
| Society of Interventional Radiology | https://www.sirweb.org/ | Practice Resources > Clinical Practice > Guidelines, Clinical Topic |
| Society of Nuclear Medicine and Molecular Imaging | https://www.snmmi.org/ | Evidence & Quality > Clinical Guidelines |
| Society of Thoracic Surgeons | https://www.sts.org/ | Resources > Clinical Practice Guidelines |

^a^Web site sections are reported in a hierarchical path with the root section listed first and subsequent subsections indicated by arrow heads (>).

^b^The American Academy of Physical Medicine and Rehabilitation affirms or endorses clinical practice guidelines created by other organizations but does not create new clinical practice guidelines.

^c^Clinical Practice Guidelines are not evidence-based

References for S1 Table

1. Kouba DJ, LoPiccolo MC, Alam M, Bordeaux JS, et al. Guidelines for the use of local anesthesia in office-based dermatologic surgery. J Am Acad Dermatol. 2016; 74(6):1201-1219.

2. AAFP Clinical Practice Guideline Manual. [cited 2017 Mar 11]. Available from: <http://www.aafp.org/patient-care/clinical-recommendations/cpg-manual.html#Introduction>.

3. American Academy of Neurology. 2011. Clinical Practice Guideline Process Manual, 2011 ed. St. Paul, MN: The American Academy of Neurology.

4. American Academy of Ophthalmology Pediatric Ophthalmology/Strabismus Panel. Preferred Practice Pattern ® Guidelines. Amblyopia. [cited 2017 Mar 17]. San Francisco, CA: American Academy of Ophthalmology; 2012. Available from: <http://www.aao.org/ppp>.

5. Rosenfeld RM, Shiffman RN, Robertson P. Clinical Practice Guideline Development Manual, Third Edition: a quality-driven approach for translating evidence into action. Otolaryngol Head Neck Surg. 2013; 148(1 Suppl):S1-55.

6. Tieder JS, Bonkowsky JL, Etzel RA, et al. Subcommittee on Apparent Life Threatening Events. Brief resolved unexplained events (formerly apparent life-threatening events) and evaluation of lower-risk infants. Pediatrics. 2016 May;137(5):e20160590.

7. Gonzalez-Campoy JM, St Jeor ST, Castorino K, et al. American Association of Clinical Endocrinologists.; American College of Endocrinology and the Obesity Society. Clinical practice guidelines for healthy eating for the prevention and treatment of metabolic and endocrine diseases in adults: cosponsored by the American Association of Clinical Endocrinologists/the American College of Endocrinology and the Obesity Society. Endocr Pract. 2013; 19 Suppl 3:1-82.

8. Methodology Manual and Policies From the ACCF/AHA Task Force on Practice Guidelines. American College of Cardiology.[cited 2017 Feb 10]. Available from: <http://www.acc.org/guidelines/about-guidelines-and-clinical-documents/methodology>.

9. Lo BM, Carpenter CR, Hatten BW, Wright BJ, Brown MD. American College of Emergency Physicians. Clinical policy: critical issues in the evaluation of adult patients with suspected transient ischemic attack in the emergency department. Ann Emerg Med. 2016;68(3):354-70.

10. National Guideline Clearinghouse, U.S. Department of Health and Human Services, Agency for Healthcare Resesarch and Quality. Guideline Summary: Second Trimester Abortion, NGC:009900, June 2013. [cited 2017 Feb 11]. Available from: <https://www.guideline.gov/summaries/summary/46411>.

11. American College of Occupational and Environmental Medicine, Clinical Practice and Guidance Center: Methodology for ACOEM’s Occupational Medicine Practice Guidelines –2016 Revision. [cited 2017 Feb 1]. Available from: <https://www.acoem.org/practiceguidelines.aspx>.

12. Qaseem A, Snow V, Owens DK, Shekelle P; Clinical Guidelines Committee of the American College of Physicians.. The development of clinical practice guidelines and guidance statements of the American College of Physicians: summary of methods. Ann Intern Med. 2010; 153(3):194-199.

13. American College of Radiology, Practice Parameters and Technical Standards, Development and Revision Handbook, 2015. [cited 2017 Feb 28]. Available from: <http://www.acr.org/guidelines>.

14. American College of Rheumatology, Clinical Practice Guidelines. Policy and Procedure Manual for Clinical Practice Guidelines (January 2015). [cited 2017 Feb 15]. Available from: <http://www.rheumatology.org/Practice-Quality/Clinical-Support/Clinical-Practice-Guidelines>.

15. Glauser T, Shinnar S, Gloss D, et al. Evidence-Based Guideline: Treatment of Convulsive Status Epilepticus in Children and Adults: Report of the Guideline Committee of the American Epilepsy Society. Epilepsy Curr. 2016; 16(1):48-61.

16. AGA Guidelines Policies and Procedures. [cited 2017 Mar 13]. Available from: <http://www.gastro.org/guidelines-policies>.

17. American Geriatrics Society, British Geriatrics Society, and American Academy of Orthopaedic Surgeons Panel on Falls Prevention. Guideline for the prevention of falls in older persons. J Am Geriatr Soc. 2001; 49(5):664-672.

18. New Development Process for Practice Guidelines of the American Psychiatric Association. [cited 2017 Mar 13]. Available from: <http://www.psychiatry.org/psychiatrists/practice/clinical-practice-guidelines/guideline-development-process>.

19. Rupp SM, Apfelbaum JL, Blitt C, et al. Practice Guidelines for Central Venous Access: A Report by the American Society of Anesthesiologists Task Force on Central Venous Access. Anesthesiology. 2012; 116(3):539-73.

20. ASCO Guidelines Methodology Manual. [cited 2017 Mar 13]. Available from: <https://pilotguidelines.atlassian.net/wiki/display/GW/Guideline+Development+Process#GuidelineDevelopmentProcess-ReviewProcess>.

21. Sepulveda AR, Hamilton SR, Allegra CJ, et al. Molecular biomarkers for the evaluation of colorectal cancer: guideline from the American Society for Clinical Pathology, College of American Pathologists, Association for Molecular Pathology, and American Society of Clinical Oncology. J Mol Diagn. 2017;19(2):187-225.

22. Paquette I, Madhulika V, Ternent C, et al. The ASCRS Clinical Practice Guideline for the Evaluation and Management of Constipation. Dis Colon Rectum*.* 2016; 59(6): 479-92.

23. Rizzo JD, Brouwers M, Hurley P, et al. American Society of Hematology/American Society of Clinical Oncology clinical practice guideline update on the use of epoetin and darbepoetin in adult patients with cancer. Blood. 2010; 116(20): 4045-59.

24. Evidence-Based Clinical Practice Guideline: Breast Reconstruction with Expanders and Implants. American Society of Plastic Surgeons.[cited 2017 Mar 18]. Available from: <https://www.plasticsurgery.org/Documents/medical-professionals/health-policy/evidence-practice/breast-reconstruction-expanders-with-implants-guidelines.pdf>.

25. Cabrera AR, Kirkpatrick JP, Fiveash JB et al. Radiation Therapy for Glioblastoma: An ASTRO Evidence-Based Clinical Practice Guideline. Pract Radiat Oncol. 2016; 6(4): 217-25.

26. Practice Committee of the American Society for Reproductive Medicine. Uterine septum: a guideline. Fertil Steril. 2016; 106(3):530-540.

27. American Urological Association Guidelines and Policies: Standard Operating Procedure.[cited 2017 Mar 13]. Available from:  <https://www.auanet.org/education/standard-operating-procedures-overview.cfm>.

28. Congress of Neurological Surgery, Guidelines: CNS/AANS Joint Guidelines Committee. [cited 2017 Mar 13]. <https://www.cns.org/guidelines>.

29. North American Spine Society Evidence-Based Clinical Guideline for Multidisciplinary Spine Care: Diagnosis and Treatment of Adult Isthmic Spondylolisthesis. [cited 2017 Mar 13]. Available from: <https://www.spine.org/Documents/ResearchClinicalCare/Guidelines/AdultIsthmicSpondylolisthesis.pdf>.

30. Jacobi J, Bircher N, Krinsley J, et al. Guidelines for the use of an insulin infusion for the management of hyperglycemia in critically ill patients. Crit Care Med. 2012; 40(12):3251-76.

31. Wright AA, Bohlke K, Armstrong DK, et al. Neoadjuvant chemotherapy for newly diagnosed, advanced ovarian cancer: Society of Gynecologic Oncology and American Society of Clinical Oncology Clinical Practice Guideline. Gynecol Oncol*.* 2016; 143(1): 3-15.

32. Pabon-Ramos WM, Dariushnia SR, Walker TG, et al. Quality Improvement Guidelines for Percutaneous Nephrostomy. J Vasc Interv Radiol*.* 2016; 27(3):410-4.

33. Delbeke D, Chiti A, Christian P, et al. SNM/EANM Guideline for Guideline Development 6.0. J of Nucl Med Tech*.* 2012; 40(4);283-9.

34. The Society of Thoracic Surgeons Policy on the Clinical Practice Guideline Writing Process. [cited 2017 Mar 13]. Available from: <https://www.sts.org/sites/default/files/documents/Guideline%20development%20process_final%20June%2012_2013.pdf>.
