# Supplementary material for: Approval processes in evidence-based clinical practice guidelines sponsored by medical specialty societies
Source: PLoS One. 2020 Feb 12;15(2):e0229004. doi: 10.1371/journal.pone.0229004 (PMC7015697; doi:10.1371/journal.pone.0229004)
Supplement: S2 Table — (DOCX) [file pone.0229004.s002.docx]

| **S2 Table. Approval Processes for Evidence-based Clinical Practice Guidelines by Medical Specialty Societies in the Council of Medical Specialty Societies** | | | | |
| --- | --- | --- | --- | --- |
|  |  |  |  |  |
| **Medical Specialty Society** | **Source of information about approval**^a^ | **Documentation of Approval Process** | **Committee issuing final approval**^b^ | **Approval criteria** |
| American Academy of Allergy, Asthma & Immunology | No guidelines procedure manual identified and no information on guideline approval process, if any, in individual guidelines. Review process reported in multiple guidelines but committee granting final approval and criteria for approval not reported. | Not applicable | Unknown. | Unknown |
| American Academy of Dermatology (AAD) | AAD Guidelines for the use of local anesthesia in office-based dermatologic surgery | “This guideline has been developed in accordance with the American Academy of Dermatology (AAD)/AAD Association Administrative Regulations for Evidence-based Clinical Practice Guidelines (version approved August 2012), which includes the opportunity for review and comment by the entire AAD membership and final review and approval by the AAD Board of Directors.”^1^ | Board of Directors | Not specified |

| **S2 Table. Approval Processes for Evidence-based Clinical Practice Guidelines by Medical Specialty Societies in the Council of Medical Specialty Societies, continued** | | | | |
| --- | --- | --- | --- | --- |
|  |  |  |  |  |
| **Medical Specialty Society** | **Source of information about approval** | **Documentation of Approval Process** | **Committee issuing final approval** | **Approval criteria** |
| American Academy of Family Physicians (AAFP) | AAFP Clinical Practice Guideline Manual | “The SCPG [Subcommittee on Clinical Practice Guidelines], a subcommittee of the CHPS [Commission on Health of the Public and Science] that is responsible for guideline review and development, will review the final draft of the guideline. Upon approval, a recommendation is made to the full CHPS, which upon approval makes a recommendation to the AAFP Board of Directors for approval.”^2^ “The AAFP Board of Directors reviews the guideline. Any questions from the Board are addressed by the GDG [Guideline Development Group], and staff at the AAFP. Upon approval from the Board, the guideline is sent to collaborators for possible endorsement.”^2^ |  |  |
| American Academy of Hospice and Palliative Medicine (AAHPM) | Not applicable-does not produce evidence-based guidelines | Not applicable | Not applicable | Not applicable |
| American Academy of Neurology (AAN) | AAN Clinical Practice Guideline Process Manual | “When the manuscript has been accepted for publication in the Neurology® journal, AAN staff submits it to the AAN Board of Directors for approval. Requests for revision during the approval process are reviewed by the GDS [Guideline Development Subcommittee] chairs. Substantive revisions may require reapproval by the GDS and Practice Committee.”^3^ | Board of Directors | Not specified |
| American Academy of Ophthalmology (AAO) | AAO Preferred Practice Pattern ® Guidelines. Amblyopia | “Approved by Board of Trustees September 25, 2012”^4^ | Board of Trustees | Not specified |
| **S2 Table. Approval Processes for Evidence-based Clinical Practice Guidelines by Medical Specialty Societies in the Council of Medical Specialty Societies, continued** | | | | |
|  |  |  |  |  |
| **Medical Specialty Society** | **Source of information about approval** | **Documentation of Approval Process** | **Committee issuing final approval** | **Approval criteria** |
| American Academy of Otolaryngology—Head and Neck Surgery (AAO-HNS) | AAO-HNS Clinical Practice Guideline Development Manual, Third Edition. | “The executive summary and full-text guideline are distributed to the board members for review, comment, and approval. The summary grid of external peer review comments and their disposition may also be included.”  “Because the document is based on evidence, any substantive changes requested by an oversight body (e.g., the organizational board of directors) must be supported and accompanied by additional evidence. The oversight body should be informed, however, that the purpose of review is not to rewrite the guideline but rather to ensure that the guideline has been developed using approved methodology and that the recommended actions are clear, appropriate, and consistent with the organization’s mission.”^5^ | Board of Directors | Fidelity to approved methodology |
| American Academy of Pediatrics (AAP) | AAP Clinical Practice Guideline on Brief Resolved Unexplained Events (Formerly Apparent Life-Threatening Events) and Evaluation of Lower-Risk Infants | “The practice guideline underwent a comprehensive review by stakeholders before formal approval by the AAP, including AAP councils, committees, and sections; selected outside organizations; and individuals identified by the subcommittee as experts in the field. All comments were reviewed by the subcommittee and incorporated into the final guideline when appropriate.”^6^ | Board of Directors^c^ | Not specified |
| **S2 Table. Approval Processes for Evidence-based Clinical Practice Guidelines by Medical Specialty Societies in the Council of Medical Specialty Societies, continued** | | | | |
|  |  |  |  |  |
| **Medical Specialty Society** | **Source of information about approval** | **Documentation of Approval Process** | **Committee issuing final approval** | **Approval criteria** |
| American Academy of Physical Medicine and Rehabilitation (AAPM&R) | Not applicable-does not produce evidence-based guidelines. Affirms or endorses guidelines produced by other groups. | Not applicable | Not applicable | Not applicable |
| American Association of Clinical Endocrinologists (AACE) | AACE, American College of Endocrinology and the Obesity Society Clinical Practice Guidelines for Healthy Eating for the Prevention and Treatment of Metabolic and Endocrine Diseases in Adults. | "This clinical practice guideline (CPG) has been reviewed and approved by the primary writers, other invited experts, the American Association of Clinical Endocrinologists (AACE) Publications and Nutrition Committees, and the AACE Board of Directors prior to submission for peer review in *Endocrine Practice*."^7^ | Board of Directors | Not specified |
| American College of Cardiology (ACC) | Methodology Manual and Policies From the ACCF/AHA Task Force on  Practice Guidelines | “BOT [Board of Trustees] and SACC [AHA Science Advisory Coordinating Committee] members may not comment or vote on clinical documents at the time of board review and approval if they have relevant RWI [Relationship with Industry] related to the document topic. Documents are approved as ACCF [American College of Cardiology Foundation] and AHA [American Heart Association] policy by a majority vote of BOT and SACC members who have no relevant RWI related to the document under consideration.”^8^ | Board of Trustees | Not specified |
| American College of Emergency Physicians (ACEP) | ACEP Critical Issues in the Evaluation of Adult Patients with Suspected Transient Ischemic Attack in the Emergency Department. | “This clinical policy was approved by the ACEP Board on June 22, 2016.”^9^ | Board of Directors | Not specified |
| **S2 Table. Approval Processes for Evidence-based Clinical Practice Guidelines by Medical Specialty Societies in the Council of Medical Specialty Societies, continued** | | | | |
|  | | | | |
|  |  |  |  |  |
| **Medical Specialty Society** | **Source of information about approval** | **Documentation of Approval Process** | **Committee issuing final approval** | **Approval criteria** |
| American College of Medical Genetics (ACMG) | Not applicable-does not produce evidence-based guidelines | Not applicable | Not applicable | Not applicable |
| American College of Obstetricians and Gynecologists (ACOG) | National Guidelines Clearinghouse synopsis of ACOG Practice Bulletin: Second-Trimester Abortion | “Practice Bulletins are validated by two internal clinical review panels composed of practicing obstetrician-gynecologists generalists and sub-specialists. The final guidelines are also reviewed and approved by the American College of Obstetricians and Gynecologists (ACOG) Executive Board.”^10^ | Executive Board | Not specified |
| American College of Occupational and Environmental Medicine (ACOEM) | Methodology for ACOEM’s  Occupational Medicine Practice Guidelines –  2016 Revision | “The ACOEM Board of Directors has approved the following organizational structure and methods for the development of recommendations for evidence-based practice contained in this update. The Board also has the opportunity to review and comment on all evidence-based guidelines prior to publication. Comments from the Board are reviewed in the same way as external review comments. However, in order to maintain editorial independence, the Board does not officially approve the Guidelines.”^11^ | None: Board approval not required | Not applicable |

| **S2 Table. Approval Processes for Evidence-based Clinical Practice Guidelines by Medical Specialty Societies in the Council of Medical Specialty Societies, continued** | | | | |
| --- | --- | --- | --- | --- |
|  |  |  |  |  |
| **Medical Specialty Society** | **Source of information about approval** | **Documentation of Approval Process** | **Committee issuing final approval** | **Approval criteria** |
| American College of Physicians (ACP) | ACP Clinical Practice Guidelines and Guidance Statements Summary of Methods | “After the Clinical Guidelines Committee reviews and approves a clinical guideline or guidance statement, these papers are then presented for final voting and approval as ACP policy to the ACP’s Board of Regents, the highest body of ACP. Simultaneously, we invite the Board of Governors, which represents members from all 50 states and territories, as well as our international members, to provide input.”^12^ | Board of Regents | Not specified |
| American College of Preventive Medicine (ACPM) | Not applicable-does not produce evidence-based guidelines | Not applicable | Not applicable | Not applicable |
| American College of Radiology (ACR) | ACR Practice Parameters and Technical Standards, Development and Revision Handbook | “The Council will be free to discuss the recommendations of the Reference Committee and either ‘adopt’, ‘not-adopt’ or “refer’ the parameters/standards.”  “By virtue of their adoption by the ACR Council, the practice parameters and technical standards are official policy statements of the ACR.”^13^ | American College of Radiology Council | Not specified |
| American College of Rheumatology (ACR) | ACR Policy and Procedure Manual for Clinical Practice Guidelines (January 2015) | “ACR funding or participation does not imply or guarantee ACR approval of the final publication or product of the project. To obtain ACR approval, guidelines must be formally reviewed by the ACR and approved by the ACR Board of Directors.”^14^ | Board of Directors | Not specified |

| **S2 Table. Approval Processes for Evidence-based Clinical Practice Guidelines by Medical Specialty Societies in the Council of Medical Specialty Societies, continued** | | | | |
| --- | --- | --- | --- | --- |
|  |  |  |  |  |
| **Medical Specialty Society** | **Source of information about approval** | **Documentation of Approval Process** | **Committee issuing final approval** | **Approval criteria** |
| American College of Surgeons (ACS) | No guidelines procedure manual identified and no information on guideline approval process, if any, in individual guidelines | Not applicable | Unknown | Unknown |
| American Epilepsy Society (AES) | AES Evidence-Based Guideline: Treatment of Convulsive Status Epilepticus in Children and Adults | “The completed evidence-based guidelines and algorithm were reviewed and approved by the American Epilepsy Society Guidelines Committee (members of which were not part of the writing group). It was also reviewed and commented on by the Council on Clinical Activities, whose comments were incorporated and subsequently approved. Following committee and council approval, it was submitted to the American Epilepsy Society Board; and after review, comments, and revisions, the guideline was approved prior to submission for publication”^15^ | Board of Directors | Not specified |
| American Gastroenterological Association (AGA) | AGA Guidelines Policies and Procedures | “The governing board reviews and approves the practice recommendations, the corresponding technical review and clinical decision support tools for publication in Gastroenterology.  The governing board will provide an "up or down" vote for approval, with editorial review of the final product only for clarity of presentation and to ensure the guideline construction process has been correctly followed.”^16^ | Governing Board | Fidelity to approved methodology |

| **S2 Table. Approval Processes for Evidence-based Clinical Practice Guidelines by Medical Specialty Societies in the Council of Medical Specialty Societies, continued** | | | | |
| --- | --- | --- | --- | --- |
|  |  |  |  |  |
| **Medical Specialty Society** | **Source of information about approval** | **Documentation of Approval Process** | **Committee issuing final approval** | **Approval criteria** |
| American Geriatrics Society (AGS) | Guideline for the Prevention of Falls in Older Persons | “This guideline was developed and written under the auspices of the American Geriatrics Society (AGS) Panel on Falls In Older Persons and approved by the AGS Board of Directors on April 5, 2001.”^17^ | Board of Directors | Not specified |
| American Medical Informatics Association (AMIA) | Not applicable-does not produce evidence-based guidelines | Not applicable | Not applicable | Not applicable |
| American Psychiatric Association (APA) | New Development Process for Practice Guidelines of the APA | “The Assembly recommends and the Board of Trustees votes to approve publication of new practice guidelines under the imprimatur of APA”^18^ | Board of Trustees | Not specified |
| American Society of Anesthesiologists (ASA) | Practice Guidelines for Central Venous Access: A Report by the ASA Task Force on Central Venous Access | “Approved by the ASA House of Delegates on October 19 2011.”^19^ | House of Delegates | Not specified |
| American Society of Clinical Oncology (ASCO) | ASCO Guidelines Methodology Manual | “ASCO Guidelines are reviewed and approved by the Clinical Practice Guidelines Committee (CPGC) before final submission to JCO [Journal of Clinical Oncology].”^20^ | Clinical Practice Guidelines Committee | Not specified |

| **S2 Table. Approval Processes for Evidence-based Clinical Practice Guidelines by Medical Specialty Societies in the Council of Medical Specialty Societies, continued** | | | | |
| --- | --- | --- | --- | --- |
|  |  |  |  |  |
| **Medical Specialty Society** | **Source of information about approval** | **Documentation of Approval Process** | **Committee issuing final approval** | **Approval criteria** |
| American Society for Clinical Pathology (ASCP) | Molecular Biomarkers for the Evaluation of Colorectal Cancer: Guideline From the American Society for Clinical Pathology, College of American Pathologists, Association for Molecular Pathology, and American Society of Clinical Oncology | “Each organization instituted a review process to approve the guideline. The ASCP assigned the review of the guideline to a Special Review Panel.”^21^ | Special Review Panel | Not specified |
| American Society of Colon and Rectal Surgeons (ASCRS) | The ASCRS Clinical Practice Guideline for the Evaluation and Management of Constipation | “The Clinical Practice Guidelines Committee is composed of Society members who are chosen because they have demonstrated expertise in the specialty of colon and rectal surgery...The primary authors reviewed all of the English language articles and studies in adults, systematic reviews, and meta-analyses...Recommendations were formulated by the primary authors and reviewed by the entire Clinical Practice Guidelines Committee. The final grade of recommendation was performed using the Grades of Recommendation, Assessment, Development, and Evaluation system10 (Table 1) and approved by the entire Clinical Practice Guidelines Committee.”^22^ | Clinical Practice Guidelines Committee | Not specified |

| **S2 Table. Approval Processes for Evidence-based Clinical Practice Guidelines by Medical Specialty Societies in the Council of Medical Specialty Societies, continued** | | | | |
| --- | --- | --- | --- | --- |
|  |  |  |  |  |
| **Medical Specialty Society** | **Source of information about approval** | **Documentation of Approval Process** | **Committee issuing final approval** | **Approval criteria** |
| American Society of Hematology (ASH) | ASH/ASCO [American Society of Clinical Oncology] clinical practice guideline update on the use of epoetin and darbepoetin in adult patients with cancer | “The ASCO/ASH Update Committee was charged with reviewing evidence from the systematic review and making revisions to the guideline recommendations as warranted. The guideline was submitted to Journal of Clinical Oncology and Blood for peer review. The guideline was reviewed and approved by the entire Update Committee, ASCO's Clinical Practice Guidelines Committee, ASH's Committee on Practice, ASH's Subcommittee on Quality of Care, the ASCO Board of Directors, and the ASH Executive Committee.”^23^ | Executive Committee | Not specified |
| American Society of Nephrology (ASN) | Not applicable-does not produce evidence-based guidelines | Not applicable | Not applicable | Not applicable |
| American Society of Plastic Surgeons (ASPS) | Evidence-Based Clinical Practice Guideline: Breast Reconstruction with Expanders and Implants | “After the peer review process, the guideline draft was reviewed and modified by the Post-Mastectomy Expander/Implant Breast Reconstruction Guideline Work Group to address peer review comments. The final guideline was approved by the ASPS Executive Committee during its March 2013 meeting.”^24^ | Executive Committee | Not specified |
| American Society for Radiation Oncology (ASTRO) | Radiation Therapy for Glioblastoma: An ASTRO Evidence-Based Clinical Practice Guideline | “Following integration of the feedback, the document was submitted for approval to the ASTRO Board of Directors in January 2016."^25^ | Board of Directors | Not specified |

| **S2 Table. Approval Processes for Evidence-based Clinical Practice Guidelines by Medical Specialty Societies in the Council of Medical Specialty Societies, continued** | | | | |
| --- | --- | --- | --- | --- |
|  |  |  |  |  |
| **Medical Specialty Society** | **Source of information about approval** | **Documentation of Approval Process** | **Committee issuing final approval** | **Approval criteria** |
| American Society for Reproductive Medicine (ASRM) | Practice Committee of the American Society for Reproductive Medicine. Uterine septum: a guideline | “The Practice Committee and the Board of Directors of the American Society for Reproductive Medicine have approved this report."^26^ | Board of Directors | Not specified |
| American Urological Association (AUA) | AUA Guidelines and Policies Standard Operating Procedures Overview | “Once the Guideline is approved by the Practice Guidelines Committee, it goes before the Science & Quality Council followed by the AUA Board of Directors for approval. The final Guideline is then officially published on the AUA website.”^27^ | Board of Directors | Not specified |
| Congress of Neurological Surgeons (CNS) | CNS/AANS [American Association of Neurological Surgeons] Joint Guidelines Description on Website | “The [CNS/AANS joint guidelines committee](https://www.cns.org/about-us/leadership-committees/joint-guidelines-committee) is responsible for the evaluation of internally and externally produced clinical practice guidelines of potential relevance to neurosurgical practice and recommending approval or revisions before approval by the parent organizations (CNS executive committee and the AANS board of directors).”^28^ | Executive Committee | Not specified |
| North American Spine Society (NASS) | NASS Evidence-Based Clinical Guidelines for Multidisciplinary Spine Care: Diagnosis and Treatment of Adult Isthmic Spondylolisthesis | “Once any evidence-based revisions were incorporated, the drafts were prepared for NASS Board review and approval. Edits and revisions to recommendations and any other content were considered for incorporation only when substantiated by a preponderance of appropriate level evidence."^29^ | Board of Directors | Not specified but revisions considered based on evidence |

| **S2 Table. Approval Processes for Evidence-based Clinical Practice Guidelines by Medical Specialty Societies in the Council of Medical Specialty Societies, continued** | | | | |
| --- | --- | --- | --- | --- |
|  | | | | |
|  |  |  |  |  |
| **Medical Specialty Society** | **Source of information about approval** | **Documentation of Approval Process** | **Committee issuing final approval** | **Approval criteria** |
| Society of Critical Care Medicine (SCCM) | Guidelines for the use of an insulin infusion for the management of hyperglycemia in critically ill patients | "External peer review was provided through the Critical Care Medicine editorial process, and approval was obtained by the governing board of the Society of Critical Care Medicine."^30^ | Governing Board (Council)^d^ | Not specified |
| Society of Gynecologic Oncology (SGO) | Neoadjuvant chemotherapy for newly diagnosed, advanced ovarian cancer: SGO and ASCO [American Society of Clinical Oncology] Clinical Practice Guideline | "The guideline was also reviewed and approved by the ASCO Clinical Practice Guidelines Committee, SGO Publications, and the SGO Clinical Practice Committees prior to publication."^31^ | Clinical Practice Guideline committee | Not specified |
| Society of Hospital Medicine (SHM) | Not applicable-does not produce evidence-based guidelines | Not applicable | Not applicable | Not applicable |
| Society of Interventional Radiology (SIR) | Quality Improvement Guidelines for Percutaneous Nephrostomy Clinical Practice Guidelines. | “The draft document is critically reviewed by the Standards of Practice Committee members, in either a telephone conference call or a face-to-face meeting. The finalized draft from the Committee is sent to the SIR membership for further input/criticism during a 30-day comment period. These comments are discussed by the Standards of Practice Committee, and appropriate revisions are made to create the finished standards document. Before its publication, the document is endorsed by the SIR Executive Council.”^32^ | Executive Council | Not specified |

| **S2 Table. Approval Processes for Evidence-based Clinical Practice Guidelines by Medical Specialty Societies in the Council of Medical Specialty Societies, continued** | | | | |
| --- | --- | --- | --- | --- |
|  |  |  |  |  |
| **Medical Specialty Society** | **Source of information about approval** | **Documentation of Approval Process** | **Committee issuing final approval** | **Approval criteria** |
| Society of Nuclear Medicine and Molecular Imaging (SNM) | SNM/EANM [European  Association of Nuclear Medicine] Guideline for Guideline Development | “New or revised (see section K) guidelines will be reviewed and approved by the SNM Committee on Guidelines. The guidelines will then be forwarded to the SNM Board of Directors and to the EANM Executive Committee for their final approval.”^33^ | Board of Directors | Not specified |
| Society of Thoracic Surgeons (STS) | The STS Policy on the Clinical Practice Guideline Writing Process | "Task Force members will follow the “STS Approval Process for Practice Guidelines and Other Policy Documents from the Workforce on Evidence Based Surgery,” as approved by the STS Board of Directors on January 23, 2005, and as amended from time to time. Any changes to the evidence-based recommendations that are made during the approval process, or after submission for publication, must go back to the author and the WFEBS Chair for review and verification of the evidence prior to final approval by the STS Board of Directors or Executive Committee”^34^ | Board of Directors | Not specified |

^a^Guideline procedure manuals, if they could be identified, were the first source used to determine medical specialty society approval processes. If guideline procedure manuals could not be identified, we searched for approval processes in evidence-based guidelines, starting with the most recent guidelines first.

^b^The committee issuing final approval is reported as described in the specialty society guideline procedure manual, if one existed, or in the guideline that served as evidence for documentation of guideline approval. Specialty societies have different names for their governing committees including Board of Directors, Board of Trustees, Board of Regents, Executive Committee, Executive Council, Council, Governing Board, and House of Delegates.

^c^The guideline specifies that there is “formal approval by the AAP [American Academy of Pediatrics]”. The web site of the AAP specifies, “The AAP is governed by a Board of Directors …” (<https://www.aap.org/en-us/about-the-aap/aap-leadership/Pages/default.aspx>)

^d^The guideline specifies that approval is obtained by the “governing board”. The web site of the Society of Critical Care Medicine specifies, “The Society is governed by the 23-member Council, which consists of the five members of the Executive Committee (president, president-elect, past president, secretary and treasurer), eight Specialty Section-designated seats (Anesthesiology, Clinical Pharmacy and Pharmacology, Emergency Medicine, Internal Medicine, Neuroscience, Nursing, Pediatrics and Surgery), one "collective" seat representing all other sections, six at-large seats, and three honorary members.” (<http://www.sccm.org/About-SCCM/Leadership/Pages/Council-Members.aspx>)
